# Genomics unlocks the potential of genetic resources for citrus breeding

**DOI:** 10.1270/jsbbs.24047

**Published:** 2025-02-21

**Authors:** Tokurou Shimizu, Keisuke Nonaka

**Affiliations:** 1 Institute of Fruit Tree Science, NARO, 485-6 Okitsu Nakacho, Shimizu, Shizuoka 424-0292, Japan; 2 Laboratory of Plant DNA Analysis, Kazusa DNA Research Institute, 2-6-7 Kazusa-kamatari, Kisarazu, Chiba 292-0818, Japan

**Keywords:** *Citrus*, genomes, genomic-assisted breeding, evolution, pedigree, domestication

## Abstract

The genus *Citrus* includes 162 species, but sweet oranges, grapefruits, lemons, and mandarins dominate global trade. For over 100 years, many native citrus cultivars have seen limited production. With the growing demand for new hybrid scions with higher sugar content, easy peeling, and good aroma, minor genetic resources offer potential for breeding, although low selection rates have limited their use. Recent genome sequencing of major citrus cultivars has advanced DNA marker and marker-assisted selection techniques. Additionally, Genome-wide Association Studies have identified key quantitative trait loci, and genomic prediction studies show higher prediction scores for various fruit traits. Genomic studies have clarified the origin and evolution of the genus *Citrus*, revealing that current species are hybrids of four ancient taxa (*C. maxima*, *C. medica*, *C. reticulata*, and *C. micrantha*) with several minor taxa, prompting a revision of the classification. Pedigree analysis of 67 native cultivars highlights the potential of some, like Kishu, Tachibana, Kaikoukan, and Kunenbo, as breeding parents. These advances have deepened our understanding of citrus origins, as well as the processes of domestication and diversification, revolutionizing breeding practices and enhancing the use of genetic resources in the citrus breeding program at the National Agriculture and Food Research Organization.

## Introduction

### Citrus and its impact on human society

**Fig. 1.**
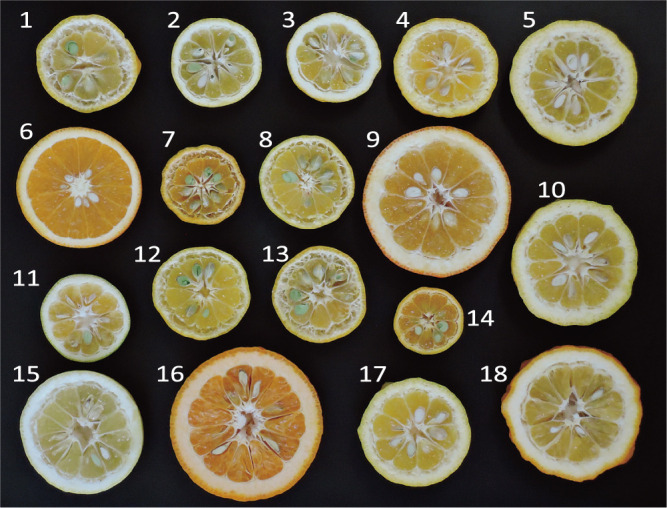
Diversity of fruit morphology in citrus genetic resources. Cross-sections of 18 Citrus Genetic Resources Collected and Maintained by NARO. Variations are observed in fruit size, rind thickness and shape, rind and flesh color, rind puffing, and degraded central area. Multiple fruits of the same variety represent different strains. 1, 13: Kabuchii (*C. kabuchi*); 4, 5, 10: Rokugatsu mikan (*C. rokugatsu*); 6: sweet orange (*C. sinensis*); 8, 12: Oto (*C. oto*); 9: sour orange (*C. aurantium*); 11, 14: Shiikuwasha (*C. depressa*); 16: Kunenbo (*C. nobilis*); 2, 3, 7, 15, 17, 18: various natural hybrids (*C.* spp.).

Citrus species are woody perennial evergreen fruit trees that belong to the Rutaceae family, subfamily Aurantioideae. They encompass a wide range of commercially important sets of cultivars, including sweet oranges, grapefruits, lemons, limes, pummelos, Yuzu, Hassaku, Natsudaidai, and mandarins such as Ponkan, Satsumas, and Clementines, as well as numerous other minor cultivars (Barry *et al.* 2020, Saunt 2000). Citrus fruits exhibit a wide diversity in size, color, and taste (Fig. 1). Poets and artists in the regions where these fruits were introduced admired their beauty and aroma (Attlee 2015, Inglese and Sortino 2019, Zhong and Nicolosi 2020). The Rutaceae family comprises about 160 genera and 1650 species (Inglese and Sortino 2019). Based on a survey of native *Citrus* genetic resources from Asia and India to the Oceania region, along with morphological characterization and discussion, Swingle placed the genera *Citrus*, *Fortunella* (Kumquat), and *Poncirus* (Karatachi; trifoliate orange), along with the genera *Eremocitrus*, *Clymenia*, and *Microcitrus*, in the subfamily Aurantioideae, tribe *Citreae*, subtribe *Citrinae* as true citrus fruit tree (Swingle and Reece 1967). Currently, the edible species belong to the genera *Citrus* and *Fortunella*, while in some regions, plants of the genus *Poncirus* are used as rootstocks. Production of citrus fruits is now spread across more than 140 countries and regions within the ‘citrus belt’, ranging from 40°N to 40°S (Inglese and Sortino 2019, Zhong and Nicolosi 2020). Citrus remains the most-produced fruit tree, with a global production of 124 million tons for fresh consumption and 23.54 million tons for processing, including juice (FAOSTAT).

Among the citrus species, the most widely produced and globally distributed are sweet oranges, lemons, grapefruits, limes, mandarins (loose rind type fruit such as Satsuma, Clementine, and Ponkan) and pummelos (Barry *et al.* 2020, Saunt 2000). In addition to their inherent aromatic qualities, these major citrus cultivars have been favored for characteristics such as the high sugar content of sweet oranges, the refreshing acidity of lemons and limes, the large fruit size of grapefruits and pummelos, and the easy peeling and eating quality of mandarins. Swingle classified groups of several varieties as a single species (Swingle and Reece 1967), while Tanaka classified each distinguishable individual variety as a separate species (Tanaka 1954, 1960). The economic benefits may be limited, but certain regions or cultures have found greater value in specific native cultivars or their mutant varieties, cherishing them as food or flavor. For example, bergamot (*C. bergamia*) is used in Earl Grey tea, makrut lime (*C. hystrix*) is used in Thai cuisine (Inglese and Sortino 2019, Saunt 2000, Swingle and Reece 1967), and Yuzu (*C. junos*) has been cultivated in Japan since at least the 8th century (Shimizu *et al.* 2019). These cultivars and their varieties have been cultivated for hundreds of years or more, and they have long been deeply intertwined with local food, culture, and customs.

### Diversity of the genus *Citrus*

The plants of the genus *Citrus* have the ability to interbreed with other species as well as with other genera, *Poncirus* and *Fortunella* to yield hybrids (Swingle and Reece 1967). Since some of these hybrids exhibit characteristics similar to those of existing types, it has been suggested that many of the native cultivars may have originated from natural hybrid seedlings. This cross-compatibility has also led to a prolonged debate about the criteria for classifying *Citrus*. Most plants in the genus *Citrus*, except for citron and pummelo, have heterogeneous genomes, and their traits can easily change through seed propagation (Caruso *et al.* 2020, Swingle and Reece 1967). Therefore, they should be maintained and propagated asexually by grafting to preserve their nature (Caruso *et al.* 2020). On the other hand, many mandarin varieties, lemons, grapefruits, sweet oranges, sour oranges, trifoliate oranges, and their hybrids produce apomictic (polyembryonic) seeds, in which embryos develop asexually from the nucellar tissue. This trait is highly valuable in citrus production and has been utilized for clonal propagation via seeds for over 1000 years (Caruso *et al.* 2020, Kepiro and Roose 2007). Mutant selection of polyembryonic seeds or as bud mutants (sports) has contributed to altering specific fruit traits (Kamatyanatt *et al.* 2021, Rauf *et al.* 2013, Roose and Williams 2007), along with induced changes in traits and disease resistance through mutagenesis by irradiation or triploid/tetraploid selection (Barry *et al.* 2020). In citrus, mutation breeding has played a role in altering specific traits of existing plants and enhancing their economic value. This approach has compensated for the difficulties of altering a particular trait through traditional crossbreeding.

### Motivations for the use of genetic resources

In the past two decades, mandarin varieties such as Satsuma, Clementines, and Ponkans, known for being easy to peel, seedless or with few seeds, and convenient to eat, have gained popularity worldwide. As a result, there is now a high demand in the market for new citrus scions that are easy to eat, are high in quality, and have fewer seeds. These are preferred over those that have hard-to-peel skins, many seeds, and susceptibility to citrus canker, which is commonly found in sweet oranges and grapefruits. Mutation breeding relies on large-scale economic cultivation and long-term exploration of characteristics in the field. However, its scope of change is limited to a specific trait. In contrast, crossbreeding is more suitable for developing new scions aimed at modifying multiple traits. Crossbreeding has been conducted in Japan, the U.S., Spain, and other countries with the goal of developing new hybrid scions that are novel and modifying existing elite cultivars. As a result, many promising hybrid scions have been released (Barry *et al.* 2020, Caruso *et al.* 2020, Saunt 2000, Shimizu 2022). In recent years, it has been pointed out that the repeated use of few superior cultivars in crossbreeding has led to a decline in the diversity of bred hybrids (Shimizu *et al.* 2016). Integrating minor cultivars into cross-breeding is anticipated to increase diversity. Nevertheless, only some have been utilized due to an extended juvenile period and a low occurrence of promising seedlings from crossed populations, which prolongs the breeding period (Shimizu 2022).

The large genetic diversity found within the genus *Citrus* hampered DNA marker development and practical application (Shimizu *et al.* 2020). Varieties of horticultural crops and their wide range of genetic diversity and characteristics, even within a single crop, have hindered genome analysis. However, breakthroughs in high-throughput and low-cost next-generation sequencing (NGS) analysis techniques have extended their use to horticultural crops, enabling the release of many reference genomes over the past decade (Chen *et al.* 2019). For citrus, the release of reference genome sequences has facilitated the development of highly accurate and transferable codominant markers, promoting their application in classification, genetic analysis, and marker-assisted selection (MAS) (Shimizu 2020, Shimizu *et al.* 2020). Enriching gene annotations in accordance with the reference genome sequence has enabled comparison and estimation of gene functions, utilizing evidence from gene-to-trait interactions accumulated in model plants. The enhancement of genomic information and the cost reduction of NGS analysis have also facilitated the development of high-throughput genotyping platforms, assessment of genetic diversity and population structures, and the use of genome-wide association studies (GWASs) to identify genes involved in a target trait and useful DNA markers for MAS, contributing to efficient fruit tree breeding (Badenes *et al.* 2016).

Most *Citrus* plants are diploid with a basic chromosome number of 2*n* = 2*x* = 18. The genome sequencing analysis of *Citrus* has progressed significantly over the past decade, with whole genome sequences being sequenced for sweet orange, Clementine, pummelo, Satsuma, lemon, and others (Di Guardo *et al.* 2021, Shimizu *et al.* 2017, Wang *et al.* 2017, 2021, Wu *et al.* 2018, Xu *et al.* 2013). The decoded genome sequence resources of citrus plants allow for highly accurate DNA marker development, GWAS, and genomic prediction, which promote breeding efficiency (Shimizu 2020, Shimizu *et al.* 2020). Recently, comparative genomic analyses of multiple *Citrus* genomes have revealed the origin, domestication, and diversification processes of the genus *Citrus*, and the genealogy of individual species has also been elucidated in detail (Curk *et al.* 2016, Shimizu *et al.* 2016, Talón *et al.* 2020, Wang *et al.* 2017, 2021, Wu *et al.* 2014, 2018, 2021, Zhong and Nicolosi 2020). With the accumulation of genomic data in citrus, comparative analysis of sequences among species and genera has become feasible, and pangenome studies have also been launched for citrus. The pangenome study was initiated as a comparative genomics approach to find solutions through big data analysis of multiple genomes, initially focusing on Streptococcus (Tettelin and Medini 2020). It has since been applied to plants with larger genome sizes as genome sequencing costs have declined (Chen *et al.* 2019, Hu *et al.* 2024). The construction of pangenome and databases has also been conducted in citrus. These pangenome tools and databases allow for estimating the origin of primordial ancestors and polyembryony in the genus *Citrus*, which has contributed to understanding the evolution of citric acid metabolic pathways (Huang *et al.* 2023, Wang *et al.* 2017, 2018).

As with many crops and horticultural plants, the use of diverse genetic resources has been limited in citrus. However, recent advances in genomic research have provided new insights into the hidden potential of genetic resources with new methods and technologies that enable their use. Based on the latest findings, accumulated data, and newly developed high-precision tools, this review introduces advances in genomic studies concerning the origin and evolution of the genus *Citrus*, the classification and genealogy of cultivars, and the identification of critical genes. Furthermore, it presents a new breeding approach that enables the rapid development of novel, high-quality fruit by intensively using genomics-assisted breeding techniques with minor genetic resources revealed through genealogy.

## Genetic resources and domestication history of citrus

### Domestication history of the genus *Citrus*

With very few actual fossils or archaeological finds of citrus plants or their ancestor plants, early studies of the origin and domestication of citrus had to rely on guesses based on observations of existing plants, descriptions in historical records, and their geographic distribution. A historical Chinese document Yugong written around 2000 BC describes two citrus types, large and small (possibly mandarin and pummelo), and Sacred writings in Sanskrit around the year 800 BC refer to citron and lemon (Chinese Society of Citriculture 2008, Inglese and Sortino 2019, Nicolosi 2007, Tolkowsky 1938, Zhong and Nicolosi 2020). These descriptions suggest that citron and lemon were the main species in India, while in China, mandarins and several types were probably used in these regions (Nicolosi 2007, Zhong and Nicolosi 2020). Early cultivation began in several of these areas in the BCE period. Alexander the Great and others later introduced citron to the Western Mediterranean from Greece via Persia, Palestine, and Egypt, but it was not considered edible at the time (Attlee 2015, Inglese and Sortino 2019, Zhong and Nicolosi 2020). Commercial production of citrus fruits began gradually in the 9th century with the introduction of lemons and bitter oranges (sour oranges) to Italy. Vasco da Gama brought sweet oranges to Europe from India no later than 1498, and then commercial production began (Attlee 2015, Inglese and Sortino 2019, Zhong and Nicolosi 2020). Citrus plants were not native to the Americas originally but were introduced by Columbus in 1493. Other citrus plants were introduced in the 16th century, and oranges were planted in Florida and South Carolina in 1656 (Inglese and Sortino 2019, Swingle and Reece 1967). Oranges, lemons, and pummelos were introduced to Africa *via* Europe, and sweet oranges were introduced to South Africa in 1654. In Australia, three native citrus relatives, *Microcitrus australis* (syn. *C. australis*; Australian lime), *M. australasica* (syn. *C. australasica*; finger lime), and *Eremocitrus glauca* (syn. *C. glauca*; desert lime) grow naturally but had not been produced commercially. Sweet oranges were introduced in 1788 for commercial production purposes. The subsequent introduction of various cultivars from India and China to the West, spreading to the Americas, resulted in natural hybrids such as Clementines, limes, and grapefruits, initiating their commercial production (Inglese and Sortino 2019, Swingle and Reece 1967). Recent genome-level analysis of sweet oranges also revealed their propagation from southern China to the United States via Europe (Wang *et al.* 2021). With the growing interest in citrus in Europe after the 16th century, Giovanni de Ferrari published the first book on citrus and their health benefits in 1646, and he also discussed attempts at classification and hybridization within its pages (Ferrari 1646). Later, Linnaeus first systematically classified the genus *Citrus* (Linné 1753); however, this classification was incomplete as mandarins had not yet been introduced to Europe. In 1811, Galesio published his book, which introduced the cultivars and cultivation of citrus, as well as their improvement through crossbreeding. This publication laid the foundation for understanding their classification and the subsequent development of the European citrus industry (Gallesio 1811).

### Diversification of citrus in Japan

Tachibana (*C. tachibana*) and Shiikuwasha (*C. depressa*) have long been considered the native citrus species in Japan (Tanaka 1954). According to the Nihon Shoki (Chronicles of Japan), ‘Tokijikunokakunokonomi’, as described, was brought to Japan by Tajimamori from ‘Tokoyokoku’ by order of the Emperors in AD 70 (Prince Toneri 720), and it is considered the first citrus introduced to Japan. ‘Tokijikunokakunokonomi’ has traditionally been regarded as Tachibana, but other opinions suggest it may be other species, such as Kishu (*C. kinokuni*) or sour oranges (*C. aurantium*), with differing opinions also regarding the actual date of introduction (Shimizu *et al.* 2019, Tanaka 1954). Surveys of past literature indicate that Tachibana, Yuzu, Kishu mandarin, and Kunenbo were already cultivated in Japan by the Nara era (AD 710–794) (Shimizu *et al.* 2019). These plants were believed to have been introduced to Japan *via* trade or diplomatic exchanges with mainland China or Southeast Asia, and spread to regions west of the Kanto area shortly thereafter (Shimizu *et al.* 2019). Pummelos, sour oranges, and Koji (*C. leiocarpa* hort. ex Tanaka) began to be cultivated in various regions across Japan starting in the Heian era (8th century) and had already spread by the Sengoku era (16th century). Once society became stable in the Edo era (1603–1867), the long-term maintenance of trees in the same location promoted natural crossing events. Subsequently, many cultivars were selected from these natural hybrids in various regions (Shimizu *et al.* 2019). During the Edo era (1603–1868), the publication of ‘Yamato Honzo’, the first Japanese book on herbalism, sparked interest and led to surveys of new plants (Kaibara 1709). After the publication of ‘Yamato Honzo’, many herbals were published introducing the features of Satsuma, Girimikan, Sudachi, Kabosu, Hassaku, Natsudaidai (Natsumikan), and others (Shimizu *et al.* 2019). Both sweet oranges and lemons were introduced to Japan in the late Edo period (1776), but their commercial production started later in the Meiji period (1868–1912). Though Iyo, Andokan, and Yamabuki were discovered and cultivated throughout the Meiji era, few remain today (Shimizu *et al.* 2019, Tanaka 1946, 1954).

### Diversity and classification of the native citrus cultivars

The classification of native citrus plants is intimately associated with recognizing their origins. Gallesio described some citrus hybrids that developed by crossing resembling known native cultivars and suggested their origin as hybrids (Gallesio 1811). Swingle also considered many citrus species to have hybrid origins based on his own crossing studies. According to these observations, he classified the genus *Citrus* into the wild subgenus *Papeda*, which placed 6 species, and the cultivated subgenus *Citrus*, which placed 10 species, a total of 16 species (Swingle and Reece 1967). Swingle’s classification system is distinctive because it assigns a single species name to a group of varieties derived from natural hybrids due to their morphological similarity. However, using a single species name for multiple cultivars becomes inconvenient when trying to specify a particular one. In addition, some criticized the classification of grapefruit as a single species as inconsistent, since it was already known as a natural hybrid (Gmitter 1995, Hodgson 1961, Tanaka 1954). While Tyozaburo Tanaka agreed with Swingle that a significant number of native varieties were natural hybrids, he also emphasized the importance of apparent natural hybrids in horticulture, placing 162 species within the genus *Citrus* (Tanaka 1954, 1960). Tanaka’s system simplified the relationship between cultivars and species, making it convenient to represent a cultivar by its species name. However, his classification system has also faced criticism for deviating from standard scientific classification. Several classification systems were proposed afterward, but establishing clear criteria based on morphological traits proved difficult.

### Resources for the collection and conservation of citrus genetic resources

A wide range of variations in fruit characteristics is observed among citrus fruits, including fruit size and shape, skin and flesh color, bitterness, acid content, sugar content, time of acid reduction and color break, skin thickness and peelability, smoothness of fruit surface, aroma, flesh quality, and number of seeds (Caruso *et al.* 2020, Swingle and Reece 1967). In addition to variations in fruit characteristics, certain citrus types exhibit differences in stress resistance, including tolerance to soil salinity and low temperatures, as well as resistance to diseases such as Alternaria, Black spot, Citrus canker, citrus tristeza virus (CTV), *Mal Secco*, Scab, and others (Caruso *et al.* 2020). Usually, each trait is inherited as an innate characteristic. However, some traits are acquired through mutations, such as the color change in blood oranges and grapefruits, and mutations affecting early fruit acid reduction. Most genetic resources are produced on a small scale for private use and receive little attention. On the other hand, certain species, such as the Australian finger lime, which produces fruits with fewer edible parts, have recently gained popularity through social media due to their visually attractive, unique fruit shape and caviar-like flesh, making them appealing as decorations and seasonings. The Australian finger lime has recently been recognized as a resource resistant to citrus greening disease, also known as Huanglongbing disease (HLB) (Wang and Trivedi 2013). HLB is a bacterial disease caused by *Candidatus Liberibacter* spp. It has spread globally and caused extensive damage to the citrus industry, particularly in the U.S. This resistance is currently being analyzed at the genetic level (Weber *et al.* 2022). Using genetic resources in breeding has been anticipated as a means to enhance diversity and increase farmers’ income by reducing environmental impact and production costs through improved quality and disease resistance. Additionally, changes in society and the environment may reveal new benefits for these genetic resources. On the other hand, many of the genetic resources described by Tanaka and Swingle have lesser economic importance, and there is a risk of their unnoticed disappearance due to environmental changes or the development of farming and residential areas. This concern is shared among agricultural scientists, including those of *Citrus* (Salgotra and Chauhan 2023). Consequently, efforts to collect and conserve citrus genetic resources have long been undertaken, especially in citrus-producing countries.

A considerable portion of citrus cultivars hold a highly heterozygous genome, making them difficult to maintain and propagate by seedling. Additionally, citrus seeds are quite labile to desiccation and quickly lose their germination ability. Therefore, vegetative propagation in the field or in pots is mandatory. Consequently, maintaining citrus genetic resources requires larger fields and more extensive long-term economic support and efforts than maintaining seed plants. The Villa Medici di Castello and Boboli Gardens in Italy still keep the Medici’s collection of citrons, sour oranges, lemons, and other citrus plants collected since the 16th century. These are the earliest collections of citrus genetic resources in Europe. They were preserved in glass greenhouses, which were extremely expensive and precious in the 17th century, demonstrating the wealth and power of the Medici family (Attlee 2015).

The efforts to collect and maintain citrus genetic resources have been undertaken independently by each country. In 2023, a report on the global collection and conservation of citrus genetic resources in citrus-producing countries worldwide was summarized and published based on an extensive survey conducted with the support of The Global Crop Trust (Volk *et al.* 2023). According to this report, 11 sites in Brazil, China, the Philippines, Iran, Italy, France, Japan, Spain, the U.S., and Vietnam have been working to collect and preserve citrus genetic resources and provide a public database (Volk *et al.* 2023). These countries conserve citrus genetic resources *ex situ* in the field, but significant risks of losing them due to environmental changes are unavoidable. Thus, international collaboration aimed at permanently maintaining genetic resources through sharing has also been discussed.

## Insights into the domestication and diversification process of the genus *Citrus* revealed by genome analysis

### The process of diversification

**Fig. 2.**
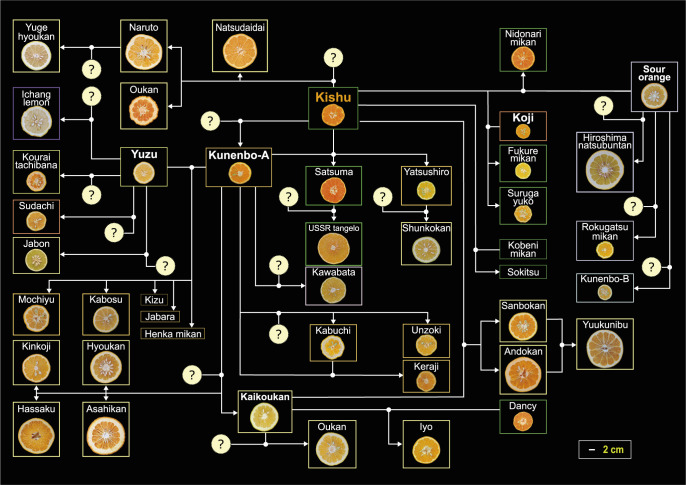
Pedigree of native citrus varieties, focusing on Kishu Mikan, Kunembo, Yuzu, Kouji, Daidai, and Kaikoukan and their progeny. The color of the rectangles surrounding the varieties corresponds to the cytoplasmic genotype (Shimizu *et al.* 2016); ‘?’ denotes unidentified unknown parents.

The occurrence of hybrid seedlings that resembled native varieties in early breeding attempts suggested that the current citrus species had differentiated and diversified through repeated hybridization of a few ancestral species. DNA marker analysis technology has enabled the classification of the genus *Citrus* and speculation on the pedigree of species and cultivars (Nicolosi 2007). The first citrus genomes were released from 2012 to 2013 (Wu *et al.* 2014, Xu *et al.* 2013), facilitating the development of highly transferable codominant DNA markers. This advancement contributed to a better understanding of citrus taxonomy and estimation of its pedigree (Shimizu *et al.* 2020). These studies have revealed that most modern citrus species are derived from four basic ancestral taxa: native citron (*C. medica*), pummelo (*C. maxim*a), mandarin (*C. reticulata*), and papeda (*C. micrantha*). They have expanded their diversity through repeated hybridization (Table 1). Using carefully selected highly accurate SNP markers, Curk and colleagues revealed the phylogenetic origin of limes, lemons, sour oranges, and sweet oranges, which emerged from the hybridization of four basic taxa (Curk *et al.* 2016). Although the origins of the hybrid cultivars of mandarin and pummelo have long been unknown, Shimizu *et al.* (2016) revealed the pedigrees of 67 cultivars, including Satsuma, Iyo, Kabosu, Hassaku, and Natsumikan, through analysis using highly accurate SSR markers (Fig. 2, Table 2). The revealed pedigrees suggest that in citrus, a few key cultivars—such as Kishu (*C. kinokuni*), Kunenbo (*C. nobilis*), and Yuzu (*C. junos*)—were initially introduced over a wide area of Japan and cultivated in the same fields for hundreds of years. This would facilitate repeated hybridization and promote the emergence of chance seedlings. The revealed pedigree also indicates that Satsuma, Iyo, Hassaku, Natsudaidai, and other acid citrus cultivars were selected from a single or several crosses. It suggests that one or two rounds of crossing are sufficient to expand diversity and select promising individuals. Furthermore, identifying Tachibana, which produces small, seedy, and sour fruits, and Kaikoukan, which has been overlooked due to its average fruit quality, as the parents of unique cultivars such as Hyuganatsu and Iyo, suggests their potential as breeding parents (Shimizu 2022, Shimizu *et al.* 2016).

## The origin and evolution of the genus *Citrus* and proposals for a new classification system

### The origin and evolution of the genus *Citrus*

Genome sequence and structural analyses suggest that the primordial citrus species originated in the southeastern Himalayan region, encompassing eastern Assam (India), western Yunnan (China), and Myanmar, approximately 8 to 7 million years ago (Talón *et al.* 2020, Wu *et al.* 2018). The primordial species are assumed to have gradually expanded their habitats and diversified through tectonic isolation and adaptation to climate change (Talón *et al.* 2020). The primordial citrus differentiated into wild ancestral taxa, citron (*C. medica*), mandarins (*C. reticulata*), pummelos (*C. maxima*), makrut lime (*C. hystrix*), and papeda (*C. micrantha*), in addition to several true citrus species. Interspecific hybridization among these species and the resulting hybrids from random crossings have been a driving force in the evolution of native citrus species to the present day, leading to an expansion of their diversity. Tachibana and Shiikuwasha have long been considered endemic to Japan, but their origin and relationship to neighboring Asian citrus were unknown. Comparative genomic analysis of 69 East Asian and other Asian citrus species suggests that the diffusion of *C. reticulata* and *C. ryukyuensis* ined. in the region from Taiwan to the Nansei Islands resulted in hybridization, giving rise to these two species (Wu *et al.* 2021).

### New classification system

Based on the understanding of the origin and genealogy of citrus plants revealed by genome-wide studies, new classification systems that rely on the latest classification rules (Turland *et al.* 2018) have been proposed, replacing the previous system that relied on morphological characteristics. According to the current understanding of the evolutionary process of the genus *Citrus*, Ollitrault *et al* (2020). defined six basic taxa as *C. cavaleriei* H. Lév. ex Cavalerie, *C. maxima* (Burm.) Merr., *C. medica* L., *C. micrantha* Wester, *C. reticulata* var. *austera* Swingle, and *C. reticulata* var. *tachibana* ined. Wu *et al.* (2018) proposed that *C. reticulata* Blanco include only nonintrogressed mandarins, *C. reticulata* var. *austera* classified by Swingle, and *C. tachibana* (Mak.) Tanaka classified by Tanaka, based on WGS analysis (Ollitrault *et al.* 2020, Wu *et al.* 2018). Subsequently, they proposed nine hybrid taxa (*C.* × *amblycarpa*, *C.* × *aurantiifolia*, *C.* × *aurantium* L., *C.* × *latifolia*, *C.* × *limon*, *C.* × *limonia*, *C.* × *lumia*, *C.* × *microcarpa*, and *C.* × *pseudolumia* ined.) that consist of citrus plants recognized for their hybrid origin (Ollitrault *et al.* 2020). Among these, six hybrid taxa, *C.* × *amblycarpa*, *C.* × *aurantiifolia*, *C.* × *aurantium*, *C.* × *limonia*, *C.* × *lumia*, and *C.* × *microcarpa*, are regarded as bispecific admixtures of two ancestors: *C. micrantha*/*C. reticulata*, *C. micrantha*/*C. medica*, *C. reticulata*/*C. maxima*, *C. reticulata*/*C. medica*, *C. medica*/*C. maxima*, and kumquat/*C. reticulata*, respectively. Additionally, two hybrid taxa, *C.* × *limon* and *C.* × *pseudolumia*, are regarded as the trispecific admixture of three ancestors: *C. reticulata*/*C. maxima*/*C. medica*, and *C. maxima*/*C. medica*/*C. micrantha*, respectively. Similarly, *C.* × *latifolia* is regarded as a tetraspecific admixture of four ancestors, *C. reticulata*/*C. maxima*/*C. medica*/*C. micrantha*. Of these mentioned hybrid taxa, seven (*C.* × *aurantiifolia*, *C.* × *aurantium* L., *C.* × *latifolia*, *C.* × *limon*, *C.* × *limonia*, *C.* × *lumia*) include several hybrid cultivars. For example, economically important cultivars such as grapefruit, sweet orange, Clementine, and Satsuma, are classified as varieties of *C.* × *aurantium* along with sour orange, Willowleaf mandarin, Kishu mandarin, King mandarin, Dancy, Temple tangor, and other minor mandarins.

On the other hand, Mabberley (2022) pointed out the difficulty of classifying groups of hybrids with complex parentage based on the ‘hybrid formula’ of the International Code of Nomenclature (ICN) (Turland *et al.* 2018). He then proposed following customary practices with cultivated plants and using cultivar groups according to the International Code of Nomenclature for Cultivated Plants (Brickell *et al.* 2016, Mabberley 2022). Based on that proposal, he defined 11 wild citrus species (*C. australasica* F. Muell., *C. australis* (Mudie) Planchon, *C. cavaleriei* H.Lév. ex Cavalerie, *C. glauca* (Lindl.) Burkill, *C. hystrix* DC., *C. japonica* Thunb., *C. maxima* (Burm.) Merr., *C. medica* L., *C. reticulata* Blanco, *C. ryukyuensis* ined., and *C. trifoliata* L.) and classified Australian lime types, kumquats, pummelos, citrons, wild mandarins, and trifoliate oranges separately. Furthermore, he defined 14 hybrid species (*C.* × *amblycarpa* (Hassk.) Ochse, *C.* × *aurantiifolia* (Christm.) Swingle, *C.* × *aurantium* L., *C.* × *floridana* (J.W. Ingram & H.E. Moore) Mabb., *C.* × *georgiana* Mabb., *C.* × *insitorum* Mabb., *C.* × *junos* (Makino) Tanaka, *C.* × *latifolia* (Yu. Tanaka) Tanaka, *C.* × *limon* (L.) Osbeck, *C.* × *microcarpa* Bunge, *C.* × *oliveri* Mabb., *C.* × *otaitensis* (Risso & Poit.) Risso, *C.* × *tachibana* (Makino) Tanaka, and *C.* × *virgata* Mabb.). He classified natural hybrid types (Nasnaran, Limes, Lemons, Tachibana, Yuzu, Calamondin, and mandarin hybrids including orange, grapefruit, Clementine, Satsuma, tangelo, tangerine, and tangor), along with crossbred cultivars (limequat, citrangequat, citrange, citrumelo, and hybrid limes) into these hybrid taxa. Whether natural hybrids and crossbred cultivars should be considered the same is debatable, but assigning a common authorized name to all cultivars is beneficial for both growers and distributors.

## Refinement of gene mining through genome-level analysis

Recent advancements in citrus genome sequencing have significantly accelerated the identification of alleles associated with target traits, facilitated the development of linkage markers, and enabled the identification of causative genes. These improvements have enhanced breeding efficiency and the selection of target traits through MAS (Shimizu and Iwata 2024). Comparative genome sequence analysis studies have identified genes related to essential traits, such as citric acid metabolism, and have estimated their evolutionary processes (Huang *et al.* 2023, Wang *et al.* 2018). A genome-wide resequencing analysis has been conducted to identify genes responsible for the precocious flowering of trifoliate orange (Zhang *et al.* 2016). Additionally, an analysis of the fruit maturation transcriptome was conducted on three pure species (citron, pure mandarin, and pummelo) and four domesticated genetic admixtures (sour orange, sweet orange, lemon, and domesticated mandarin). This analysis revealed significant differences between their transcriptomes, particularly in the admixed regions (Borredá *et al.* 2022).

*Ruby*, the causal gene for the blood orange color mutation, was identified through genomic analysis of sweet orange mutant lines (Butelli *et al.* 2012). Furthermore, the gene responsible for polyembryony (*CitRWP*), which is involved in asexual reproduction in the genus *Citrus*, was identified through genome sequencing and GWAS analysis of 100 monoembryonic and polyembryonic accessions (Wang *et al.* 2017, Xu *et al.* 2021), and another study confirmed its function through the expression analysis in transgenic plants (Shimada *et al.* 2018). Both *Ruby* and *CitRWP* are mutations caused by genomic insertions of retrotransposons, which induce phenotypic variation. In general, a mutation occurring in one of the many vegetatively propagated clonal individuals is typically lost from the population rapidly and does not become fixed. In contrast, beneficial mutations and those favoring survival in citrus became fixed through hybridization. These mutations were then spread within the population over the long term through asexual methods such as grafting and nucellar seedlings. Genomics-based studies enabled the identification of mutated genes that emerged from these processes and facilitated their use in breeding.

To conduct extensive genome-wide genotyping analysis, Hiraoka and colleagues developed 1.4 million SNPs and a 54,000 SNP Citrus Genotyping Array for a wide range of citrus accessions, based on whole-genome re-sequencing data from 41 diverse citrus and closely related accessions (Hiraoka *et al.* 2024). Furthermore, researchers demonstrated the usefulness of genome-wide genotype data collected from various accessions or hybrids for identifying genomic regions associated with target traits through GWAS analysis. They also showed how fruit traits can be predicted using genomic prediction approaches (Shimizu 2020). Given the high heterozygosity observed in citrus hybrids, it indicates that the combination of alleles is the main factor driving the diversity observed in populations bred from closely related crosses (Shimizu and Iwata 2024), rather than the presence or absence of specific gene sets targeted by pangenome analysis (Chen *et al.* 2019, Hu *et al.* 2024, Tettelin and Medini 2020). On the other hand, the ability of the genus *Citrus*, along with the genera *Poncirus* and *Fortunella*, to cross with each other suggests that the presence or absence of specific members within a gene family is also a significant factor in raising diversity in crossed hybrids between genetically distant species or cultivars. To date, comparative genome analysis has focused on specific key gene family members, including WRKY, CYP, gibberellic acid metabolism, citrate metabolism, and several morphogenesis genes (Huang *et al.* 2023, Shimizu and Iwata 2024). Pan-genomic studies have recently commenced for plants, fruit trees, and citrus (Chen *et al.* 2019, Hu *et al.* 2024, Huang *et al.* 2023, Liu *et al.* 2022, Wang *et al.* 2018, Wu *et al.* 2021), with further investigations expected to elucidate the role of gene family members and the contribution of allele combinations in enhancing diversity.

## Genomics for the use of genetic resources in breeding

### Current issues in citrus breeding

Current citrus breeding, including the program conducted at the National Agricultural Research Institute (NARO), requires the rapid development of highly novel new scions with superior quality from a single cross. The juvenile period of citrus trees, which lasts 7 to 10 years when grown from seedlings, can be shortened to approximately 5 years through grafting (Yamada *et al.* 1979, Yoshida 1980). However, continuous evaluations over several years are necessary, as the fruit from the first set often does not show the full performance. Therefore, it has taken approximately 22 years to select a promising individual from sown seeds and to register it after repeated evaluations of its traits.

Fruit tree breeding, including citrus, involves evaluating over 20 characteristics such as fruit quality, disease/abiotic stress resistance, productivity, yield stability, flowering, and tree vigor over multiple years (Badenes and Byrne 2012, Caruso *et al.* 2020, Raveh *et al.* 2020, Shimizu 2020, 2022). However, selecting seedlings that excel across all these multiple criteria simultaneously will drastically reduce the number of promising individuals (Shimizu 2022). Suppose *W* is the number of individuals grafted and *P* is the number of individuals evaluated and selected; then the acquisition rate of promising seedlings (*R*) is expressed as *R* = *P*/*W*. At NARO, the acquisition rate (*R*) is approximately 0.02%. In addition, conventional breeding based on phenotypic evaluation requires an extended period to assess fruit traits in the field. Improving traits gradually through successive generations significantly lengthens the breeding period. Increasing the size of the hybrid population to select promising seedlings that emerge at low frequency within a single generation effectively shortens the breeding period. (Shimizu 2022). In fruit tree breeding, seedlings are typically grafted onto rootstocks in the field to shorten the juvenile period and promote uniform growth. Consequently, the number of seedlings grafted onto rootstocks in the field determines the actual breeding scale. Selecting candidate seedlings before grafting improves breeding efficiency and increases the number of promising seedlings obtained per generation (Shimizu 2022). Fruit tree breeding has traditionally relied on a limited number of elite cultivars that excel for their high fruit quality, productivity, and other desirable traits. While this approach has helped increase the frequency of promising seedlings per generation, the repeated use of these elite parents has led to a converning reduction in the overall genetic diversity within breeding population (Shimizu *et al.* 2016).

### Current challenges in expanding diversity

The elucidated genealogy of citrus indicates that a single generation is often sufficient for selecting novel high-quality even from crosses of minor cultivars such as Tachibana and Kaikoukan, which have not been previously utilized in breeding. However, minor cultivars often possess multiple undesirable characteristics simultaneously, such as small fruit size, tough peel, low sugar content, high seed count, difficulty chewing due to fibrous texture, and an unpleasant taste and off-flavors (Fig. 1). Many of these fruit characteristics are quantitative traits, making it uncommon for individuals to exceed the selection threshold. Consequently, using minor cultivars for crossbreeding makes it challenging to select promising seedlings that meet the selection criteria within a single generation. This often necessitates either drastically scaling up the number of seedlings for breeding or conducting selection over several generations. However, achieving the former is difficult in conventional breeding, while the latter leads to a prolonged breeding period.

### Breeding using genomics-assisted selection

Breeding fruit trees, including citrus, requires extensive time and considerable effort for trait evaluation of hybrid populations in the field. Additionally, the challenges associated with expanding population sizes limit the detailed analysis of quantitative trait loci (QTLs). The high genomic heterozygosity in citrus also hinders the application of QTLs detected in a specific population to other populations. This constraint often restricts the use of the detected QTLs in MAS. With the decline in the cost of NGS and genome-wide genotyping analysis, GWAS and genomic prediction have been practically applied in fruit tree breeding (Badenes *et al.* 2016, Iwata *et al.* 2016, Mathiazhagan *et al.* 2021, van Nocker and Gardiner 2014), and their utility in citrus breeding has also been confirmed (Minamikawa *et al.* 2017, Shimizu 2020). According to a GWAS analysis conducted with citrus populations for 17 fruit traits, Minamikawa *et al.* (2017) identified genomic regions strongly associated with traits such as fruit weight (Weight), fruit appearance (Appear), fruit shape (Shape), fruit hardness (FruH), pericarp color (ColorP), pericarp smoothness (SmoothP), ease of peeling (Peeling), aroma intensity (Aroma), flesh color (ColorF), flesh hardness (FleH), juiciness (Juicy), locule membrane firmness (FirmLM), number of seeds (Seed), bitterness (Bitter), taste (Taste), sugar content (Brix), and acidity (Acid), with correlations exceeding the significance level. Among the 11 detected traits, 2 (Weight and Acid) were continuous variables, while the remaining 9 (FruH, ColorP, Peeling, ColorF, FleH, Juicy, FirmLM, Seed, and Bitter) had discrete rank values. These results indicate the availability of GWAS for evaluating fruit traits that are often represented using rank values based on visual or sensory assessment. Furthermore, a genomic prediction trial of the 17 fruit traits in the citrus population demonstrated that 5 (Weight, FruH, ColorP, Peeling, ColorF, and FirmLM) exhibited high predictive accuracy (Pearson’s correlation coefficient, *r* ≥ 0.7), while 9 traits (Shape, SmoothP, Aroma, FleH, Juicy, Seed, Bitter, Taste, Brix, and Acid) showed moderate accuracy (0.3 ≤ *r* < 0.7) (Minamikawa *et al.* 2017).

While the prediction accuracy varied by trait, the relatively high accuracy observed in traits evaluated using visual assessment scores indicated the utility of genomic prediction. This suggests that genomic prediction can be effectively used not only for continuous numerical data but also for traits assessed through visual assessment. On the other hand, the prediction accuracy for Appear (*r* < 0.3) was low among the evaluated traits. This trait represents a categorical value of fruit appearance scored by visual assessment, and incorrect association of each rank with the appearance might be the reason for the low prediction accuracy.

Increasing the number of individuals used to construct the prediction model in genomic prediction analysis effectively improved prediction accuracy. This trend was more pronounced for traits with initially low prediction accuracy, confirming that using a larger number of admixed populations for model construction is effective in improving trait prediction accuracy. Minamikawa and colleagues also demonstrated that the fruit weight of large-fruit group pummelos could be predicted with high accuracy, showing correlation coefficients ranging from 0.74 to 0.89, using a model constructed solely with small fruit–weight mandarins and their hybrids (Minamikawa *et al.* 2017). Contrary to this, the prediction accuracy for the fruit weight of the small-fruit group was low (*r* = 0.30 to 0.32) when using the prediction model constructed using the large-fruit group.

An analysis of the origins and genealogies of major cultivars frequently used in breeding, including sweet orange, Satsuma, Clementine, and Ponkan, demonstrated that their genomes are admixtures of ancestral pummelos and mandarins (Table 1) (Talón *et al.* 2020). The study also indicates that large fruit types, such as grapefruit, Hassaku, and Natsudaidai originated from crosses between sweet orange, Kishu mandarin, and Kunenbo with pummelo (Fig. 2, Table 2) (Shimizu *et al.* 2016, Talón *et al.* 2020). The traits of pummelo, such as its large fruit size, thick rind, and difficulty in peeling, are considered heritable, with its genome being admixed into the genomes of small-fruit hybrids. This genomic admixture is considered to contribute significantly to the accurate prediction of fruit weight. Conversely, large-fruit pummelos are assumed to contain almost no ancestral mandarin genome. The low prediction accuracy of small-fruit types using prediction models solely constructed with those large-fruit types of pummelos indicates that integrating the genotypes of hybrids that share a common origin with the target into the prediction models is a valid method to significantly improve prediction accuracy (Minamikawa *et al.* 2017). Genealogical study has revealed that minor types, which are rarely used in breeding, also share a common genetic background with those frequently used in breeding (Shimizu *et al.* 2016). These findings strongly suggest that QTL detection by GWAS and genomic prediction can achieve high accuracy by using plant samples that share a common origin, even when minor types are used as hybrid parents.

### Toward citrus breeding 2.0

MAS is a crucial tool in fruit tree breeding. By selecting individuals based on genetic markers prior to grafting, breeders can evaluate a much larger pool of promising seedlings than would be possible through field trials alone. This expansion of the breeding scale allows for greater selection intensity, as the number of acquired individuals can be significantly increased compared to traditional methods. MAS is especially valuable when physical field capacity limits the total number of individuals that can be evaluated. Integrating MAS into breeding programs enables breeders to maximize the number of promising seedlings identified in each generation, even under practical constraints.

For fruit tree breeding, where the period from sowing to field evaluation is lengthy, the cost of maintaining seedlings in the field can exceed the cost of MAS. By using MAS to early-select individuals not expected to meet the target trait thresholds, and grafting only the presumed high-performing seedlings, breeders can both expand the overall breeding scale and decrease the cost per selected promising individual. The population of individuals exceeding the selection criteria is usually low, but a further decline is assumed to be inevitable when genetic resources that are less available for use in breeding are used as parents. Consequently, several generations of selection are necessary when using such genetic resources as parents, resulting in a lengthy breeding process. MAS and trait prediction by genome-wide genotyping over a large number of seedlings, rather than phenotypic selection, enable the selection of promising individuals with low occurrence within a single generation and provide a solution to avoid the low occurrence of promising seedlings when using minor genetic resources for crosses.

Based on the systematic selection of promising seedlings through genome-wide genotyping of large populations and the identity by descent of their genomes, as shown by the genealogy of native citrus cultivars, new breeding approaches such as rebreeding, complementary breeding, and mimic breeding have been proposed (Shimizu 2022). Rebreeding aims to select new hybrids with characteristics similar to those of the target cultivars by re-crossing their parents of known cultivars, such as Satsuma, Clementine, and Iyo, and selecting individuals whose genome-wide genotypes resemble those of the target.

Complementary breeding is like rebreeding but aims to be an alternative to mutant breeding by selecting individuals whose genome-wide genotypes closely resemble those of the target cultivar after stepwise selection of traits by MAS. Mimic breeding, on the other hand, aims to select novel promising individuals from crosses of minor cultivars identified from the revealed genealogy as parents and selects candidates through MAS, rather than selecting based on a similarity of genome-wide genotypes to a specific target.

In the use of GWAS and genomic prediction for citrus breeding, improving prediction accuracy across a wide range of hybrid combinations is crucial. Increasing the number of individuals and repeated trait evaluations for QTL detection and trait prediction model building are effective in improving prediction accuracy. However, fruit investigation must be completed within short timeframes after harvest, which restricts the number of fruits for the analysis. Among trait evaluations, improvement is expected in visual evaluation, given its susceptibility to evaluators. Minamikawa and colleagues reported an effort to predict fruit hardness and peelability by using machine learning on fruit section images, aiming to improve breeding efficiency by automating trait evaluation (Minamikawa *et al.* 2022). Furthermore, association analysis of the fruit images dissected into flavedo, albedo, seed, fruit center, degraded central area, and locule number suggested an interaction among these elemental traits, providing new insights into the fruit growth process of citrus. Applications of machine learning to fruit trait evaluation benefit from increasing the amount and accuracy of data provided for building a trait prediction model by eliminating evaluator-dependent bias and increasing throughput in visual evaluations. While genome sequencing has dramatically improved the efficiency of citrus breeding, additional genomic studies have revealed the origin, domestication process, and pedigrees, highlighting the hidden potential of genetic resources for citrus breeding.

## Author Contribution Statement

TS proposed the concept of using genetic resources for breeding, and both TS and KN conducted the genomic breeding studies on which this review is based. TS prepared the manuscript draft, while KN contributed to its revision. All authors approved the manuscript for publication.

## Figures and Tables

**Table 1.** Genealogy of major citrus types with their supposed hybrid combinations

Citrus type	Origin or hybrid combination
Mandarins (Type 1)	Pure mandarins (no admixture of other taxa; *C. reticulata*)
Mandarins (Type 2)	Mandarin (Type 1) × Pummelos (*C. maxima*) e.g. sour oranges, Cleopatra, Sunki, Dancy, Willowleaf mandarin, ponkan
Mandarins (Type 3)	Mandarin (Type 2) × Pummelos (*C. maxima*) e.g. sweet orange, W. Murcott, Wilking, King mandarins
Lemon	Sour orange × citron (*C. medica*)
Rangpur lime	Mandarins (Type 1) × citron (*C. medica*)
Rough lime	Mandarins (Type 1) × citron (*C. medica*)
Clementine	Willowleaf mandarin × sweet orange
Grapefruit	Pummelo (*C. maxima*) × sweet orange
Mexican lime	*C. micrantha* × citron (*C. medica*)

Compiled from Talón *et al.* (2020).

**Table 2.** Pedigree of native citrus cultivars with their supposed hybrid combinations

Variety	Female parent	Male parent
Andokan	Kaikoukan	Kishu
Asahikan	N.D.	Kunenbo-A
Fukure mikan	Kishu	Koji
Hanayu	Yuzu	Tachibana-A
Hassaku	N.D.	Kunenbo-A
Hiroshima natsubuntan	Sour orange	N.D.
Hyuganatsu	N.D.	Tachibana-B
Ichang lemon	N.D.	Yuzu
Iyo	Kaikoukan	Dancy
Jabon	N.D.	Yuzu
Jabara	Kunenbo-A	Yuzu
Kabosu	Kunenbo-A	Yuzu
Kabuchi	Kunenbo-A	N.D.
Kaikoukan	N.D.	Kunenbo-A
Kawabata	N.D.	Kunenbo-A
Keraji	(Kunenbo-A)	(Keraji)
Kourai tachibana	Yuzu	N.D.
Kunenbo-A	N.D.	Kishu
Mochiyu	Kunenbo-A	Yuzu
Naruto	N.D.	Kishu
Natsudaidai	N.D.	Kishu
Nidonari mikan	Kishu	Sour orange
Oogonkan	N.D.	Tachibana-C
Oukan	N.D.	Kishu
Sanbokan	Kaikoukan	Kishu
Satsuma	Kishu	Kunenbo-A
Sudachi	N.D.	Yuzu
Suruga yuko	Kishu	Koji
Yatsushiro	Kunenbo-A	Kishu
Yuukunibu	(Sanbokan)	(Andokan)
Unzoki	Kunenbo-A	N.D.

Compiled from Shimizu *et al.* (2016). N.D.: not identified. Varieties in parentheses are those for which the male and female varieties have not been determined.
